# Oxidative Aromatization, Cytotoxic Activity Evaluation and Conformational Study of Novel 7-aryl-10, 11-dihydro-7H-chromeno [4, 3-b]quinoline-6, 8(9H, 12H)-dione Derivatives.

**Published:** 2014

**Authors:** Radineh Motamedi, Abbas Shafiee, Mohammad Reza Rezai, Omidreza Firuzi, Najmeh Edraki, Ramin Miri

**Affiliations:** a *Department of Chemistry, Payame Noor University, Delijan, Iran.*; b *Pharmaceutical Sciences Research Center, Faculty of Pharmacy, Tehran University of Medical Sciences, Tehran 14174, Iran.*; c *Medicinal and Natural Products Chemistry Research Center, Shiraz University of Medical Sciences, Shiraz, Iran.*

**Keywords:** Oxidative aromatization, Chromeno [4, 3-*b*]quinolone, Cytotoxicity, Conformational analysis

## Abstract

In the present work, novel 7-aryl-10, 11-dihydro-7*H*-chromeno [4, 3-*b*]quinoline-6, 8(9*H*, *12H*)-dione derivatives were synthesized by oxidation of 7-aryl-8, 9, 10, 12-tetrahydro-*7H*-chromeno[4, 3-*b*]quinoline-6, 8-diones in the presence of silica sulfuric acid/NaNO_2_ with yields of 64-74%. Cytotoxic activity of synthesized compounds was assessed on three different human cancer cell lines (K562, LS180, and MCF-7). Synthesized compounds showed moderate cytotoxic activities. The most active one apeared to be 2e, containing a methoxy group on the meta position of phenyl ring (IC_50 _range in different cell lines: 11.1–55.7 µM). Furthermore; comparison of the cytotoxic activity of these novel oxidized derivatives with non-oxidized counterparts revealed that oxidation of dihydropyridine ring to pyridine, improves the activity especially in LS180 cell line. Conformational analysis revealed that some conformational aspects of oxidized derivatives such as orientation of C_7_-aryl substitute were clearly different from non-oxidized ones.

## Introduction

1, 4-Dihydropyridine (DHP) compounds are well known as calcium channel modulators and have emerged as one of the most important classes of drugs for the treatment of cardiovascular diseases (1). Although the DHP nucleus has been particularly well explored as L-type calcium channel modulator, DHP is a privileged structure or scaffold that can, when appropriately decorated, interact with diverse receptors and possess a variety of biological activities (2). 

Previous studies have demonstrated the cytotoxic and anticancer activity of some 1, 4-DHPs derivatives (3-5). Moreover, the results of different studies indicate that 1, 4-DHP derivatives have significant inhibitory effects on MDR in cancer cell lines (6-8). 

On the other hand, synthetic and naturally occurring coumarine derivatives are one of the most promising scaffolds in medicinal chemistry (9-11). In addition, different coumarine derivatives such as 4-hydroxycoumarine and 7-hydroxycoumarine derivatives demonstrated cytotoxic and antitumoral properties (12-13).

We have previously synthesized and evaluated the cytotoxic activity of some novel heteroanalogues of fused DHPs with the features of 1,4-DHPs and 4-hydroxycoumarins named as “chromeno[4, 3-*b*]quinoline or 7-aryl-8,9,10,12-tetrahydro-7*H*-chromeno[4, 3-*b*]quinoline-6,11-dione derivatives” 1 (14). Some of these derivatives showed moderate cytotoxic capacity and at the same time very low calcium channel antagonist activity, an undesirable effect when these compounds are used as antitumoral agents. These findings prompted us to further optimize this structure for design of more potent and specific cytotoxic agents. Armed with our experience and our interest in pharmacological properties and especially cytotoxic and DNA-intercalating activity of polycondensed heterocyclic compounds such as 1,8-acridinone and benzopyrano[3, 2-c]chromene-6, 8-dione derivatives (15-18), we were persuaded to aromatize these newly synthesized 1,4-DHPs (1) in order to obtain new pyridine derivatives with higher cytotoxic effect. We also examined some structural properties by means of computational conformational analysis.

## Experimental

*Chemistry*

All chemicals and solvents used in this study were purchased from Merck (Darmstadt, Germany) and Sigma-Aldrich (San Louis, MO, USA). Melting points were determined on a Kofler hot stage apparatus and are uncorrected. The IR spectra were obtained on a Shimadzu 470 spectrophotometer (potassium bromide disks).

^1^H-NMR spectra were measured using a Bruker FT-500 spectrometer, and chemical shifts are expressed as d (ppm) with tetramethylsilane as internal standard. The mass spectra were run on a Finnigan TSQ-70 spectrometer at 70 eV. Merck silica gel 60 F254 plates were used for analytical TLC; column chromatography was performed on Merck silica gel (70–230 mesh). Yields were calculated for purified products and were not optimized.

*Typical Procedure for synthesis of* *7-Aryl-10, 11-dihydro-9H-chromeno[4,3-b]quinoline-6,8-dione* *(2)*

Compounds 1 (5.4 mmol), sodium nitrite (16 mmol, 1.1 g) and silica sulfuric acid (1.6 g) and silica (1.07 g) were refluxed in chloroform (100 mL) for 8-12 h. The mixture was cooled to room temperature and was filtered. The filtrate was evaporated to dryness under reduced pressure, and the crude product was purified by short column chromatography to give 2 (Table 1).

*7-(2-Methylphenyl)-10, 11-dihydro-6H-chromeno[4, 3-b]quinoline-6, 8-dione (2a).*

Yellowish solid 1.36 g (yield 71%), mp = 210-212 °C, R_f _= 0.70 (2:8 EtOAc/Petrolum Ether).

 ν_max_ cm^-1^(KBr): 2922, 2848 (C-H aliphatic), 1754 (C = O ester), 1691 (C = O ketone), 757 (C-H bending).

^1^HNMR (CDCl_3_), δ (ppm): 2.02 (s, 3H, CH_3_ ), 2.24 (qn, 2H, *J* = 6.6 Hz, H_10_), 2.66 (t, 2H, *J* = 6.6 Hz, H_9_), 3.38 (t, 2H, *J* = 6.6 Hz, H_11_), 6.81 (d, ^1^H, *J* = 7.0 Hz, H_15_ ), 7.23 (t, ^1^H, *J* = 7.0 Hz, H_17_), 7.28-7.35 (m, 3H, H_16_, H_18, _H_4_), 7.40 (t, ^1^H, *J* = 8.0 Hz, H_2_), 7.61 (t, ^1^H, *J* = 8.0 Hz, H_3_), 8.68 (d, ^1^H, *J* = 8.0 Hz, H_1_).

^13^CNMR (CDCl_3_), δ: 20.01, 20.98, 34.61, 40.13, 115.13, 116.90, 118.83, 124.61, 124.89, 125.58, 126.09, 127.44, 127.69, 129.26, 133.33, 134.24, 137.84, 153.43, 154.13, 156.39, 157.92, 169.58, 196.14.

 MS : *m/z* (%), 355 (M^+^, 44), 340 (44), 327 (37), 299(100), 271(37), 151(50), 126 (50), 114 (63), 100 (44), 87 (25).

Anal. Calcd for C_23_H_17_NO_3_: C, 77.73; H, 4.82; N, 3.94. Found: C, 77.40 ; H, 4.56; N, 3.95. 

*7-(3-Methylphenyl)-10, 11-dihydro-6H-chromeno[4, 3-b]quinoline-6, 8-dione (2b).*

White solid, 1.22 g (yield 64%), mp = 172-174 °C, R_f _= 0.73 (2:8 EtOAc/Petrolum Ether).

ν_max_ cm^-1^(KBr): 2848 (C-H aliphatic), 1754 (C=O ester), 1691(C=O ketone), 757 (C-H bending).

^1^HNMR (CDCl_3_), δ (ppm): 2.24 (qn, 2H, *J* = 6.6 Hz, H_10_), 2.39 (s, 3H, CH_3_ ), 2.67 (t, 2H, *J* = 6.5 Hz, H_9_), 3.36 (t, 2H, *J* = 6.5 Hz, H_11_), 6.91 (m, 2H, H_14_, H_16_ ), 7.24 (d, ^1^H, *J* = 7.5 Hz, ), 7.30 (d, ^1^H, *J* = 8.0 Hz, H_4_), 7.33 (t, ^1^H, *J* = 7.5 Hz, H_17_), 7.39 (t, ^1^H, *J* = 8.0 Hz, H_2_), 7.60 (t, ^1^H, *J* = 8.0 Hz, H_3_), 8.67 (d, ^1^H, *J* = 8.0 Hz, H_1_).

^13^CNMR (CDCl_3_), δ: 20.93, 21.64, 34.56, 40.33, 115.08, 116.83, 118.80, 123.34, 124.56, 125.67, 126.11, 126.78, 127.74, 128.41, 133.25, 133.40, 137.35, 153.41, 153.86, 156.60, 158.13, 169.29, 196.39. 

MS : *m/z* (%), 355 (M^+^, 48), 340 (38), 265 (38), 149 (86), 121 (100), 105 (23), 92 (35), 71 (35), 57 (42), 43 (50). 

Anal. Calcd for C_23_H_17_NO3: C, 77.73; H, 4.82; N, 3.94. Found: C, 77.80; H, 4.85; N, 3.97. 

*7-(4-Methylphenyl)-10, 11-dihydro-6H-chromeno[4, 3-b]quinoline-6, 8-dione (2c).*

White solid, 1.30 g (yield 68%) mp = 241-243 °C, R_f_= 0.74 (2:8 EtOAc/Petrolum Ether).

ν_max_ cm^-1^(KBr): 3033 (C-H aromatic), 2853 (C-H aliphatic), 1750 (C=O ester), 1698 (C=O ketone), 759 (C-H bending).

^1^HNMR (CDCl_3_), δ (ppm): 2.24 (qn, 2H, *J* = 6.6 Hz, H_10_), 2.46 (s, 3H, CH_3_ ), 2.68 (t, 2H, *J* = 6.6 Hz, H_9_), 3.37 (t, 2H, *J* = 6.3 Hz, H_11_), 7.02 (d, 2H, *J* = 8.0 Hz, H_15_, H_17_), 7.27 (d, 2H, *J* = 8.0 Hz, H_14_, H_18_), 7.32 (d, ^1^H, *J* = 8.2 Hz, H_4_), 7.41 (t, ^1^H, *J* = 8.2 Hz, H_2_), 7.61 (t, ^1^H, *J* = 8.2 Hz, H_3_), 8.68 (d, 1H, *J* = 8.2 Hz, H_1_). 

^13^CNMR (CDCl_3_), δ: 20.94, 21.57, 34.54, 40.33, 115.19, 116.86, 118.84, 124.56, 126, 11, 126.15, 127.94, 128.75, 133.24, 134.93, 137.19, 153.42, 153.89, 156.67, 158.25, 169.27, 196.58. 

MS : *m/z* (%), 355 (M^+^, 100), 340 (31), 327 (25), 299 (19), 127 (13). 

Anal. Calcd for C_23_H_17_NO3: C, 77.73; H, 4.82; N, 3.94. Found: C, 77.68 ; H, 4.78; N, 3.90. 

*7-(2-Methoxyphenyl)-10, 11-dihydro-6H-chromeno[4, 3-b]quinoline-6, 8-dione(2d).*

White solid, 1.42 g (yield 71%), mp = 180-182 °C, R_f _= 0.69 (2:8 EtOAc/Petrolum Ether).

ν_max_ cm^-1^(KBr): 3056 (C-H aromatic), 2852 (C-H aliphatic), 1749 (C=O ester), 1693 (C=O ketone), 753 (C-H bending).

^1^HNMR (CDCl_3_), δ (ppm): 2.25 (qn, 2H, *J* = 6.7 Hz, H_10_), 2.69 (t, 2H, *J* = 6.7 Hz, H_9_), 3.38 (t, 2H, *J* = 6.7 Hz, H_11_), 3.74 (s, 3H, OCH_3_ ), 6.90 (d, ^1^H, *J* =6.0 Hz, H_15_ ), 6.89 (d, ^1^H, *J* =6.0 Hz, H_18_ ), 7.00 (m, ^1^H, H_17_), 7.31 (d, ^1^H, *J* = 8.2 Hz, H_4_), 7.41 (t, ^1^H, *J* =6.0 Hz, H_16_ ), 7.43 (t, ^1^H, *J* =8.2 Hz, H_2_ ), 7.61 (t, ^1^H, *J* = 8.2 Hz, H_3_), 8.68 (d, ^1^H, *J* = 8.2 Hz, H_1_).

^13^CNMR (CDCl_3_), δ: 20.97, 34.48, 40.06, 55.67, 110.39, 115.60, 116.82, 118.97, 120.73, 124.49, 126.04, 126.76, 127.40, 128.07, 129.21, 133.08, 153.32, 153.51, 153.87, 155.85, 158.13, 169.18, 196.28. 

MS : *m/z* (%), 371 (M^+^, 38), 340 (100), 120 (31), 91 (31), 75 (25). 

Anal. Calcd for C_23_H_17_NO_4_: C, 74.38; H, 4.61; N, 3.77. Found: C, 74.32 ; H, 4.58; N, 3.70. 

*7-(3-Methoxyphenyl)-10, 11-dihydro-6H-chromeno[4, 3-b]quinoline-6, 8-dione (2e).*

White solid, 1.32 g (yield 66%), mp = 155-157 °C, R_f _= 0.67 (2:8 EtOAc/Petrolum Ether). 

ν_max_ cm^-1^(KBr): 3060 (C-H aromatic), 2835 (C-H aliphatic), 1755 (C=O ester), 1692 (C=O ketone), 766 (C-H bending).

^1^HNMR (CDCl_3_), δ (ppm): 2.25 (qn, 2H, *J* = 6.5 Hz, H_10_), 2.68 (t, 2H, *J* = 6.5 Hz, H_9_), 3.37 (t, 2H, *J* = 6.5 Hz, H_11_), 3.82 (s, 3H, OCH_3_ ), 6.67 (s, ^1^H, H_14_ ), 6.72 (d, ^1^H, *J* = 7.5 Hz, H_18_), 6.98 (d, ^1^H, *J* = 7.5 Hz, H_16_), 7.31 (d, ^1^H, *J* = 8.0 Hz, H_4_), 7.37-7.42 (m, 2H, H_17_, H_2_), 7.61 (t, ^1^H, *J* = 8.0 Hz, H_3_), 8.68 (d, ^1^H, *J* = 8.0 Hz, H_1_).

^13^CNMR (CDCl_3_), δ: 20.91, 34.56, 40.29, 55.13, 112.49, 112.56, 115.02, 116.86, 118.76, 124.57, 126.11, 127.62, 128.97, 133.29, 133.89, 139.26, 153.41, 153.91, 155.96, 157.97, 159.33, 169.35, 196.17.

MS : m/z (%), 371 (M^+^, 44), 340 (37), 341 (50), 196 (63), 120 (38), 91 (100), 43 ( 50).

Anal. Calcd for C_23_H_17_NO_4_: C, 74.38; H, 4.61; N, 3.77. Found: C, 74.33; H, 4.56; N, 3.68. 

*7-(4-Methoxyphenyl)-10, 11-dihydro-6H-chromeno[4, 3-b]quinoline-6, 8-dione(2f).*

White solid, 1.50 g (yield 75%), mp = 185-187 °C, R_f _= 0.71 ( 2:8 EtOAc/Petrolum Ether).

ν_max _cm^-1^(KBr): 2854(C-H aliphatic), 1747(C=O ester), 1688(C=O ketone), 761(C-H bending).

^1^HNMR (CDCl_3_), δ (ppm): 2.24 (qn, 2H, *J* = 6.6 Hz H_10_), 2.69 (t, 2H, *J* = 6.5 Hz, H_9_), 3.37 (t, 2H, *J* = 6.4 Hz, H_11_), 3.88 (s, 3H, OCH_3_ ), 7.00 (d, 2H, *J* = 8.7 Hz, H_15_, H_17_), 7.05 (d, 2H, *J* = 8.7 Hz, H_14_, H_18_), 7.32 (d, ^1^H, *J* = 8.2 Hz, H_4_), 7.41 (t, ^1^H, *J* = 8.2 Hz, H_2_), 7.62 (t, ^1^H, *J* = 8.2 Hz, H_3_), 8.68 (d, ^1^H, *J* = 8.2 Hz, H_1_). 

^13^CNMR (CDCl_3_-d), δ: 20.94, 34.54, 40.38, 55.11, 113.51, 115.29, 116.84, 118.85, 124.56, 126.12, 127.73, 128.11, 129.89, 130.03, 133.23, 153.40, 153.89, 156.37, 158.33, 169.25, 196.70.

MS : m/z (%), 371 (M^+^, 11), 289 (18), 149 29), 121 (32), 85 (63), 71 (88), 57 (100), 43 (95). 

Anal. Calcd for C_23_H_17_NO_4_: C, 74.38; H, 4.61; N, 3.77. Found: C, 74.43 ; H, 4.59; N, 3.69. 

*7-(2-Cholorophenyl)-10, 11-dihydro-6H-chromeno[4, 3-b]quinoline-6, 8-dione (2g).*

White solid, 1.33 g (yield 66%), mp = 253-255 °C, R_f_= 0.65 (2:8 EtOAc/Petrolum Ether). 

ν_max_ cm^-1^(KBr): 3068 (C-H aromatic), 2854 (C-H aliphatic), 1744 (C=O ester), 1690 (C=O ketone), 762 (C-H bending).

^1^HNMR (CDCl_3_), δ (ppm): 2.27 (qn, 2H, *J* = 6.5 Hz, H_10_), 2.69 (t, 2H, *J* = 6.5 Hz, H_9_), 3.41 (t, 2H, *J* = 6.5 Hz, H_11_), 7.03 (d, ^1^H, *J* = 7.8 Hz, H_18_), 7.27-7.43 (m, 4H, H_2_, H_4_, H_16_, H_17_), 7.50 (d, ^1^H, *J* = 7.8 Hz, H_15_), 7.62 (t, ^1^H, *J* = 7.8 Hz, H_3_), 8.70 (d, ^1^H, J = 7.8 Hz, H1). 

^13^CNMR (CDCl_3_), δ: 20.88, 34.55, 39.89, 114.97, 116.94, 118.75, 124.68, 126.08, 126.68, 127.09, 127.18, 128.92, 130.77, 133.42, 133.90, 137.28, 152.92, 153.38, 154.26, 157.99, 169.69, 196.94.

MS : *m/z* (%),377 (M^+^+2, 33), 375 (M^+^, 100), 340 (25), 187 (13), 127 (13), 113 (13), 100 (10), 87 (5).

Anal. Calcd for C_22_H_14_ClNO_3_: C, 70.31; H, 3.75; N, 3.73. Found: C, 70.40 ; H, 3.69; N, 3.70. 

*7-(3-Cholorophenyl)-10, 11-dihydro-6H-chromeno[4, 3-b]quinoline-6, 8-dione (2h).*

White solid, 1.37 g (yield 68%), mp = 188-190 °C, R_f_= 0.64 (2:8 EtOAc/Petrolum Ether).

ν_max_ cm^-1^(KBr): 3018 (C-H aromatic), 2853 (C-H aliphatic), 1744 (C=O ester), 1691 (C=O ketone), 757 (C-H bending).

^1^HNMR (CDCl_3_), δ (ppm): 2.23-2.28 (qn, 2H, *J* = 6.5 Hz, H_10_), 2.67-2.70 (t, 2H, *J* = 6.5 Hz, H_9_), 3.39-3.40 (t, 2H, *J* = 6.5 Hz, H_11_), 7.02 (d, ^1^H, *J* = 7.0 Hz, H_16_ ), 7.10 (s, ^1^H, H_14_), 7.30 (d, ^1^H, *J* = 8.0 Hz, H_4_), 7.37-7.42 (m, 3H, H_2_, H_17_, H_18_), 7.62 (t, ^1^H, *J* = 8.0 Hz, H_3_), 8.68 (d, ^1^H, *J* = 8.0 Hz, H_1_).

^13^CNMR (CDCl_3_) δ: 20.85 (C_10_), 34.57 (C_11_), 40.24 (C_9_), 114.86 (C_7_), 116.88 (C_4_), 118.64 (C_1a_), 124.59 (C_16_), 124.72 (C_2_), 126.15 (C_1_), 126.27 (C_14_), 127.26 (C_7a_), 127.62 (C_17_), 129.16 (C_18_), 133.49 (C_3_), 133.90 (C_13_), 139.79 (C_6a_), 153.38 (C_12a_), 154.04 (C_4a_), 154.48 (C_15_), 158.07 (C_6_), 169.64 (C_11a_), 196.08 (C_8_).

MS : m/z (%), 377 (M^+^+2, 10), 375 (M^+^, 30), 340 (30), 319 (27), 289 (14), 227 (44), 127 (44), 120 (100), 100 (64), 87 (32), 74 (36). 

Anal. Calcd for C_22_H_14_ClNO_3_: C, 70.31; H, 3.75; N, 3.73. Found: C, 70.38; H, 3.56; N, 3.67. 

*7-(4-Cholorophenyl)-10, 11-dihydro-6H-chromeno[4, 3-b]quinoline-6, 8-dione (2i).*

White solid, 1.41 g (yield 70%), mp = 281-283 °C, R_f _= 0.66 (2:8 EtOAc/Petrolum Ether). 

ν_max_ cm^-1^(KBr): 2851 (C-H aliphatic), 1745 (C=O ester), 1691 (C=O ketone), 753 (C-H bending).

^1^HNMR (CDCl_3_), δ (ppm): 2.25 (qn, 2H, *J* = 6.6 Hz H_10_), 2.68 (t, 2H, *J* = 6.6 Hz, H_9_), 3.38 (t, 2H, *J* = 6.6 Hz, H_11_), 7.05 (d, 2H, *J* = 8.3 Hz, H_14_, H_18_), 7.33 (d, ^1^H, *J* = 8.2 Hz, H_4_), 7.41(d, ^1^H, *J* = 8.2 Hz, H_2_), 7.43 (d, 2H, *J* = 8.3 Hz, H_15_, H_17_), 7.62 (t, ^1^H, *J* = 8.2 Hz, H_3_), 8.68 (d, ^1^H, *J* = 8.2 Hz, H_1_).

^13^CNMR (CDCl_3_), δ: 20.87, 34.56, 40.28, 114.85, 116.90, 118.62, 124.70, 126.16, 126.25, 127.64, 128.29, 133.47, 133.88, 136.46, 153.39, 154.07, 155.15, 158.08, 169.55, 196.34.

MS : *m/z* (%), 377 (M^+ ^+2, 10), 375 (M^+^, 29), 289 (100), 265 (58), 237 (64), 121 (100), 85 (44), 71 (59), 57 (80).

Anal. Calcd for C_22_H_14_ClNO_3_: C, 70.31; H, 3.75; N, 3.73. Found: C, 70.28 ; H, 3.70; N, 3.70. 

*7-(3-Nitrophenyl)-10, 11-dihydro-6H-chromeno[4, 3-b]quinoline-6, 8-dione (2j).*

White solid, 1.45 g (yield 70%), mp = 161-163 °C, R_f _= 0.64 (2:8 EtOAc/Petrolum Ether).

ν_max_ cm^-1^(KBr): 3086 (C-H aromatic), 2849 (C-H aliphatic), 1748 (C=O ester), 1693 (C=O ketone), 1543, 1343 (N=O nitro aryl), 765 (C-H bending). 

^1^HNMR (CDCl_3_), δ (ppm): 2.28 (qn, 2H, *J* = 6.6 Hz, H_10_), 2.68 (t, 2H, *J* = 6.6 Hz, H_9_), 3.39 (t, 2H, *J* = 6.6 Hz, H_11_), 7.33 (d, ^1^H, *J* = 8.0 Hz, H_4_), 7.42-7.48 (m, 2H, H_2, _H_17_), 7.61-7.66 (m, 2H, H_3_, H_18_), 7.98 (s, ^1^H, H_14_), 8.31 (d, ^1^H, *J* = 7.75 Hz, H_16_), 8.70 (d, ^1^H, *J* = 8.0 Hz, H_1_). 

^13^CNMR (CDCl_3_δ 

MS : m/z (%), 386 (M^+^, 30), 289 (37), 167 (62.5), 149 (51), 121 (29), 77 (98), 43 (100).

Anal. Calcd for C_23_H_17_NO_3_: C, 77.73; H, 4.82; N, 3.94. Found: C, 77.69 ; H, 4.80; N, 3.86. 

*7-(4-Nitrophenyl)-10, 11-dihydro-6H-chromeno[4, 3-b]quinoline-6, 8-dione (2k).*

White solid, 1.39 g (yield 67%), mp = 265-267 °C, R_f _= 0.63(2:8 EtOAc/Petrolum Ether). 

ν_max_ cm^-1^(KBr): 3072 (C-H aromatic), 2951, 2849 (C-H aliphatic), 1741 (C=O ester), 1689 (C=O ketone), 1547, 1346 (N=O nitro aryl), 765 (C-H bending).

^1^HNMR (CDCl_3_), δ(ppm): 2.25 (qn, 2H, *J* = 6.4 Hz, H_10_), 2.68 (t, 2H, *J* = 6.4 Hz, H_9_), 3.39 (t, 2H, *J* = 6.4 Hz, H_11_), 7.30 (d, 2H, *J* = 8.6 Hz, H_14_, H_18_), 7.33 (d, ^1^H, *J* = 8.2 Hz, H_4_), 7.43 (t, ^1^H, *J* = 8.0 Hz, H_2_), 7.64 (t, ^1^H, *J* = 8.0 Hz, H_3_), 8.31 (d, 2H, *J* = 8.6 Hz, H_15_, H_17_), 8.70 (d, ^1^H, *J* = 8.0 Hz, H_1_). 

^13^δ

MS : *m/z* (%),386 (M^+^, 12.5), 289(34), 265(18), 121(80), 84(100), 57(75).

Anal. Calcd for C_23_H_17_NO_3_: C, 77.73; H, 4.82; N, 3.94. Found: C, 77.80; H, 4.92; N, 3.89. 

*7-(3-Bromophenyl)-10, 11-dihydro-6H-chromeno[4, 3-b]quinoline-6, 8-dione (2l).*

White solid, 1.44 g (yiled 64%), mp = 184-186 °C, R_f _= 0.72 ( 2:8 EtOAc/Petrolum Ether).

ν_max_ cm^-1^(KBr): 3070 (C-H aromatic), 2954 (C-H aliphatic), 1748 (C=O ester), 1693 (C=O ketone), 768 (C-H bending).

^1^HNMR (CDCl_3_), δ (ppm): 2.25 (qn, 2H, *J* = 6.5 Hz, H_10_), 2.69 (t, 2H, *J* = 6.5 Hz, H_9_), 3.39 (t, 2H, *J* = 6.5 Hz, H_11_), 7.08 (d, ^1^H, *J* = 7.6 Hz, H_16_), 7.24 (s, ^1^H, H_14_), 7.32, 7.35 (m, 2H, H_4_, H_17_), 7.41 (t, ^1^H, *J* = 8.0 Hz, H_2_), 7.58 (d, ^1^H, *J* = 7.6 Hz, H_18_), 7.64 (t, ^1^H, *J* = 8.0 Hz, H_3_), 8.68 (d, ^1^H, *J* = 8.0 Hz, H_1_).

^13^CNMR (CDCl_3_), δ: 20.85, 34.58, 40.24, 114.88, 116.91, 118.64, 122.04, 124.71, 125.03, 126.16, 127.22, 128.96, 129.38, 130.52, 133.50, 140.01, 153.40, 154.08, 154.40, 158.08, 169.61, 196.06. 

MS : m/z (%), 421 (M^+ ^+2, 98), 419 (100), 340 (33), 312 (68), 169 (80), 155 (38), 127 (67), 113 (70), 100 (60), 87 ( 47). 

Anal. Calcd for C_22_H_14_BrNO_3_: C, 62.87; H, 3.36; N, 3.33. Found: C, 62.78 ; H, 3.29; N, 3.40. 

*7-(4-Bromophenyl)-10, 11-dihydro-6H-chromeno[4, 3-b]quinoline-6, 8-dione (2m).*

White solid, 1.47 g (yiled 65%), mp = 279-281 °C R_f _= 0.77 ( 2:8 EtOAc/Petrolum Ether).

ν_max_ cm^-1^(KBr): 3042 (C-H aromatic), 2922 (C-H aliphatic), 1745 (C=O ester), 1693 (C=O ketone), 771 (C-H bending). 

^1^HNMR (CDCl_3_), δ (ppm): 2.24 (qn, 2H, *J* = 6.5 Hz, H_10_), 2.67 (t, 2H, *J* = 6.5 Hz, H_9_), 3.38 (t, 2H, *J* = 6.4 Hz, H_11_), 6.98 (d, 2H, *J* = 8.3 Hz, H_14_, H_18_), 7.32 (d, ^1^H, *J* = 7.0 Hz, H_4_), 7.40 (t, ^1^H, *J* = 7.0 Hz, H_2_), 7.58 (d, 2H, *J* = 8.3 Hz, H_15_, H_17_), 7.62 (t, ^1^H, *J* = 70 Hz, H_3_), 8.67 (d, ^1^H, *J* = 7.0 Hz, H_1_). ^13^CNMR (CDCl_3_), δ: 20.87, 34.56, 40.28, 114.92, 116.91, 118.68, 124.71, 126.16, 126.26, 127.40, 127.91, 131.19, 133.48, 136.99, 153.39, 154.08, 155.10, 158.23, 169.57, 196.34.

MS : m/z (%), 421 (M^+^ +2, 11), 419 (M^+^, 11), 330 (13), 289 (100), 216 (22), 149 (20), 83 (31), 57 (76). 

Anal. Calcd for C_22_H_14_BrNO_3_: C, 62.87; H, 3.36; N, 3.33. Found: C, 62.79 ; H, 3.37; N, 3.40. 

*7-(Phenyl)-10, 11-dihydro-6H-chromeno[4, 3-b]quinoline-6, 8-dione (2n).*

Yellowish solid, 1.36 g (yield 74%), mp = 121-123 °C, R_f _= 0.79 (2:8 EtOAc/Petrolum Ether).

ν_max_ cm^-1^(KBr): 3056 (C-H aromatic), 2949 (C-H aliphatic), 1744 (C=O ester), 1693 (C=O ketone), 776 (C-H bending).

^1^HNMR (CDCl_3_), δ (ppm): 2.25 (qn, 2H, *J* = 6.6 Hz, H_10_), 2.68 (t, 2H, *J* = 6.6 Hz, H_9_), 3.38 (t, 2H, *J* = 6.6 Hz, H_11_), 7.13 (m, 2H, H_14_, H_18_ ), 7.32 (d, ^1^H, *J* = 8.0 Hz, H_4_), 7.42 (t, ^1^H, *J* = 8.0 Hz, H_2_), 7.46 (m, 3H, H_15_, H_16, _H_17_), 7.61 (t, ^1^H, *J* = 8.0 Hz, H_3_), 8.68 (d, ^1^H, *J* = 8.0 Hz, H_1_).

^13^CNMR (CDCl_3_), δ: 20.91, 34.57, 40.30, 115.06, 116.87, 118.80, 124.59, 124.75, 126.13, 126.16, 127.56, 127.92, 133.30, 138, 153.43, 153.95, 156.38, 158.19, 169.38, 196.36. 

MS : *m/z* (%), 341 (M^+^, 81), 313 (69), 289 (38), 196 (44), 120 (75), 90 (100), 76 (44). 

Anal. Calcd for C_22_H_15_NO_3_: C, 77.41, H, 4.43; N, 4.10. Found: C, 77.39; H, 4.40; N, 4.09.

*Pharmacology*

RPMI 1640, fetal bovine serum (FBS), trypsin and phosphate buffered saline (PBS) were purchased from Biosera (Ringmer, UK). 3-(4, 5-Dimethylthiazol-2-yl)-2, 5-diphenyltetrazolium bromide (MTT) was obtained from Sigma-Aldrich (Saint Louis, MO, USA) and penicillin/streptomycin was purchased from Invitrogen (San Diego, CA, USA). Doxorubicin and dimethyl sulphoxide were obtained from EBEWE Pharma (Unterach, Austria) and Merck (Darmstadt, Germany), respectively.

*Cell lines and* *Maintenance of human cell lines*

K562 (human chronic myelogenous leukemia), LS180 (human colon adenocarcinoma) and MCF-7 (human breast adenocarcinoma) cells μ_2 _and were grown in monolayer cultures_, _except for K562 cells, which were grown in suspension.

*MTT-based Cytotoxicity assay*

Cell viability following exposure to synthetic compounds was estimated by using the MTT reduction assay (19-20). K562 and LS180 cells were plated in 96-well microplates at a density of 5 × 10^4^ cells/mL (100 μL per well), While MCF-7cells were plated at densities of 3 × 10^5^. Control wells contained no drugs and blank wells contained only growth medium for background correction. After overnight incubation at 37 °C, half of the growth medium was removed and 50 μL of medium supplemented with 3 different concentrations of synthetic were added in duplicate. Plates with K562 cells were centrifuged before this procedure. Compounds were all first dissolved in DMSO and then diluted in the growth medium. The maximum concentration of DMSO in the wells was 0.5%. Cells were further incubated for 72 h and at the end of the incubation time; the medium was replaced with fresh medium containing 0.5 mg/mL of MTT. Plates were incubated for another 4 h at 37 °C. Then the formazan crystals formed in the cells dissolved in 200 μL DMSO. The optical density was measured at 570 nm with background correction at 655 nm using a Bio-Rad microplate reader (Model 680). The percentage inhibition of viability for each concentration of compound was calculated compared to the control wells and IC_50_±

*Conformational study*

The chemical structure of molecules was constructed using Hyperchem (Version 7, Hypercube Inc., http://www.hyper.com, USA). The Z-matrices of the structures were provided by the software and were then transferred to the Gaussian 98 program (21). Complete geometry optimization was performed taking the most extended conformations as starting geometries. Semi empirical molecular orbital calculations (AM_1_) of the structures were performed using Gaussian 98 program. Then we calculated some important dihedral angles of this optimized structure such as orientation of C_7_-aryl substitute and conformation of cyclohexenone ring.

## Results and Discussion

**Scheme 1:**
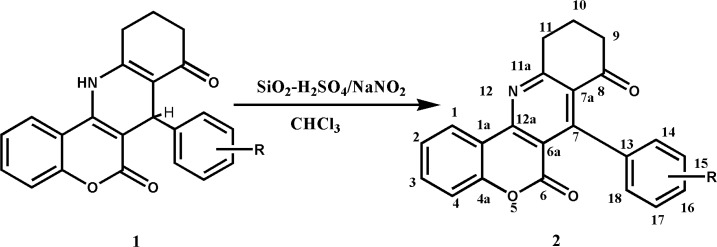
synthetic route for oxidative aromatization of chromeno[4, 3-b]quinoline

In this paper, we aromatized synthesized dihydropyridines (DHPs) (1) in the presence of oxidizing reagent, silica sulfuric acid/NaNO_2_ to corresponding new pyridine derivatives (2),** (**Scheme 1).

We first used the most common methods of oxidation by means of oxidizing agents such as Mn(IV), Ti(IV) and V(V) for the oxidation of compound 1, but all these methods proved unsuccessful. However, compound 1 could be aromatized to compound 2 using silica sulfuric acid and sodium nitrite in boiling chloroform with 64-74 yields. All reactions were completed in an appropriate time and gave only the corresponding pyridine derivatives. The results are summarized in Table 1.

**Table 1:** Reactions time and yields of oxidation reaction

**Compound**	**R**	**Time(h)**	**Yield (%)**	**MP(** ^0^ **C)**
**2a**	2-CH_3_	8	71	210-212
**2b**	3-CH_3_	8	64	172-174
**2c**	4-CH_3_	8	68	241-243
**2d**	2-OCH_3_	8	71	180-182
**2e**	3-OCH_3_	8	66	155-157
**2f**	4-OCH_3_	8	75	185-187
**2g**	2-Cl	9	66	253-255
**2h**	3-Cl	8	68	188-190
**2i**	4-Cl	8	70	281-283
**2j**	2-NO_2_	12	70	161-163
**2k**	4-NO_2_	12	67	265-267
**2l**	3-Br	8	64	184-186
**2m**	4-Br	8	65	279-281
**2n**	H	8	74	121-123

**Scheme 2:**
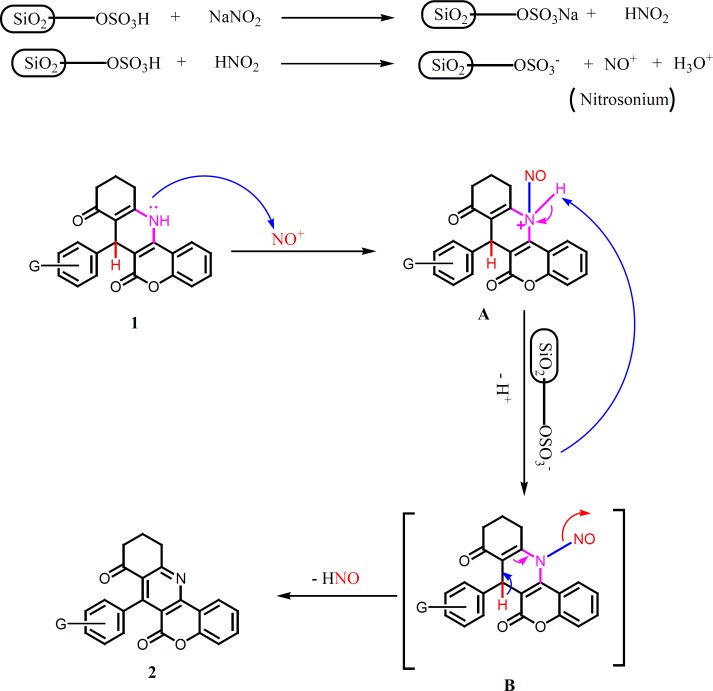
Probable Mechanism pathway for the aromatization of Chromeno [4, 3b]quinoline

The proposed mechanism involved two steps; nitrosation of DHP by nitrous acid and aromatization by losing hydrogen and NO (Scheme 2). Nitrosation reaction was performed under mild and heterogeneous conditions. In this reaction, wet SiO_2_ acts as a media and provides a heterogeneous effective surface area for in situ generation of HNO_2_.

The structures of compounds were confirmed by ^1^H NMR, ^13^CNMR and EI-MS spectra. The ^1^H NMR spectra showed the high deshielding character of aliphatic protons from 1.8-2.9 in 1 to 2.2-3.5 in 2 confirming the formation of pyridine. The protons belonging to CH and NH in dihydropyridine of compounds 1 was disappeared in compounds 2. Mass spectrum and elemental analysis clearly supports the proposed structure.

The cytotoxic activity of synthesized compounds was evaluated in three different human cancer cell lines including K562 (chronic myelogenous leukemia), LS180 (colon adenocarcinoma) and MCF-7 (breast adenocarcinoma). Data are demonstrated in Table 2. Compounds showed moderate cytotoxic activities. The most active one apeared to be 2e, containing a methoxy group on the meta position of phenyl ring, with the lowest IC_50_ values (11.1, 26.8 and 55.7 µM on LS180, MCF-7 and K562 cells, respectively). When comparing this most potent compound with the dihydropyridine derivative counterpart of our previous study (14) (1e containing methoxy group on meta position of phenyl ring) an interesting result is achieved; 1e is not active on any of these cell lines (the IC_50_ of 1e is greater than 100 µM in all three cell lines). The other compounds 2c, 2m and 2k also showed good activities. All of these compounds are more potent than the corresponding dihydropyridine derivatives especially in LS-180 cell line (the corresponding IC_50 _of 2c, 2m and 2k in LS-180 cell line is 21.9, 41.9 and 38.7 µM respectively. However, 1c, 1m and 1k were not active in this cell line and 2d, 2g and 2b compunds were inactive on all three cell lines (14).

**Table 2:** Cytotoxic activity of synthetic compounds assessed by the MTT reduction assay

	**IC**_50_ ** (µM)**		
**Compound**	**K562 cells**	**LS180 cells**	**MCF-7** **cells**
**2l**	137.1 ± 48.1	200	120.8 ± 29.8
**2n**	116.1 ± 31.7	200	90.6 ± 23.1
**2f**	200	46.9 ± 9.1	85.4 ± 21.7
**2d**	200	200	200
**2i**	67.6 ± 9.4	200	55.0 ± 20.5
**2e**	55.7 ± 26.7	11.1 ± 3.5	26.8 ± 5.9
**2c**	89.9 ± 43.1	21.9 ± 3.6	63.5 ± 13.9
**2h**	200	200	133.6 ± 30.2
**2g**	200	200	200
**2a**	132.0 ± 13.5	200	200
**2k**	147.5 ± 7.6	38.7 ± 12.1	66.4 ± 14.1
**2m**	52.5 ± 13.6	41.9 ± 6.5	43.8 ± 6.9
**2b**	200	200	200
**Cisplatin**	6.5 ± 0.5	15.5 ± 1.9	15.7 ± 9.6

Values represent the mean ± S.D. of 3 to 4 different experiments

By comparing the pyridine derivatives with the non-oxidized drivatives of our previous study (14), it can be concluded that the cytotoxic activity of some pyridine derivatives are improved especially in LS180 cell lines. Therefore the condensation and aromatization of the structure resulted in enhanced antitumoral activity in most of studied compounds. 

**Figure 1:**
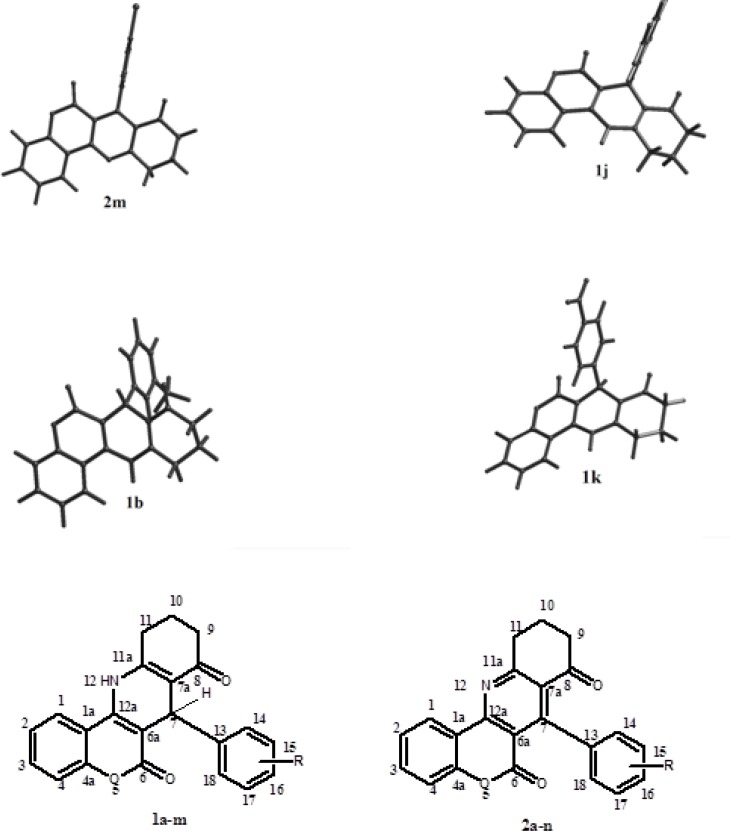
The optimized three-dimensional structural representation of six selected compounds

The optimized 3D structures of molecules (1a-n and 2a-m) were obtained by semi-empirical molecular orbital calculations (AM1). Structures of six compounds are presented in Figure. 1. The calculated dihedral angles are represented in Table 3.

**Table 3:** Geometrical features of the optimized structures of synthesized compounds

**Compound**	**R**	**pIC50** **K562 cells**	**pIC50** **LS180** ** cells**	**pIC50** **MCF7** ** cells**	**Ф1** ^a^ ** (◦)**	**Ф2** ^b^ ** (◦)**	**Ф3** ^c^ ** (◦)**
**1a**	2-CH_3_	NA^d^	NA	ND^e^	35.97	60.69	-102.43
**1b**	3-CH_3_	4.24	4.28	ND	36.69	134.21	-103.23
**1c**	4-CH_3_	4.25	4.18	ND	36.90	58.15	-102.18
**1d**	2-OCH_3_	NA	NA	ND	36.71	-123.64	-102.18
**1e**	3-OCH_3_	NA	NA	ND	44.75	51.99	-100.60
**1f**	4-OCH_3_	NA	4.21	ND	35.81	-123.56	-101.88
**1g**	2-Cl	NA	NA	ND	37.45	55.95	-102.13
**1h**	3-Cl	4.29	4.14	ND	37.41	-145.51	-104.01
**1i**	4-Cl	4.43	4.38	ND	38.59	60.22	-103.33
**1j**	2-NO_2_	4.60	4.23	ND	20.69	-63.87	101.47
**1k**	4-NO_2_	4.36	NA	ND	15.71	130.28	113.42
**1l**	3-Br	4.41	4.50	ND	23.11	-67.25	101.24
**1m**	4-Br	4.32	4.34	ND	20.25	-61.37	101.71
**2a**	2-CH_3_	3.88	NA	ND	-33.88	-92.55	-179.32
**2b**	3-CH_3_	NA	NA	ND	-52.81	-75.72	-173.63
**2c**	4-CH_3_	4.05	4.66	4.20	-47.26	-94.67	-179.92
**2d**	2-OCH_3_	NA	NA	NA	-53.46	-77.95	-150.79
**2e**	3-OCH_3_	4.25	4.95	4.57	-40.98	-101.115	-177.93
**2f**	4-OCH_3_	NA	4.33	4.07	-47.36	-96.79	-179.28
**2g**	2-Cl	NA	NA	NA	-49.20	-91.61	-173.87
**2h**	3-Cl	NA	NA	3.87	-39.98	-95.41	-178.63
**2i**	4-Cl	4.17	NA	4.26	-47.47	-93.20	-176.10
**2j**	2-NO_2_	NA	NA	NA	-37.88	-96.99	-178.68
**2k**	4-NO_2_	3.83	4.41	4.18	-41.58	-98.02	-178.94
**2l**	3-Br	3.86	NA	3.92	-43.05	-93.33	-178.58
**2m**	4-Br	4.28	4.38	4.36	-50.73	-81.2	-173.87
**2n**	H	3.94	NA	4.04	-44.02	-94.73	-179.06

^a^ Dihedral angle of cyclohexenone ring (C_7a_–C_8_–C_9_–C_10_)

^b^ Dihedral angle between phenyl and pyridine (or dihydropyridine) rings (C_6a_–C_7_–C_13_–C_14_)

^c^ Dihedral angle between phenyl and pyridine (or dihydropyridine) rings (C_12a_–C_6a_–C_7_–C_13_)

^d^ Inactive

^e ^Not determined

The cyclohexenone ring exhibited a semi-boat conformer in all studied derivatives. The “C_6a_–C_7_–C_13_–C_14_” dihedral angle reflects the orientation of aryl group at C_7_ position. As it can be seen in Table 3, the aryl group positioned at the axial coordinate especially in oxidized derivatives 2a-m (the relevant dihedral angle is 90-100^◦^). However, some deviation from axial orientation is seen in non-oxidized group 1a-n. Besides, the deviation of aryl ring from axial position is clear in less potent compounds. Moreover, it seems that the main difference of pyridine 2a-m and dihydropyridine 1a-n derivatives is the spatial orientation of C_7_-aryl ring with respect to the main structure (Table 3). The dihydropyridine series 1a-n show approximately antiplanar position with respect to four main ring structure (the relevant dihedral angle is 90-100^◦^). While in pyridine series 2a-m, the aryl ring adopt synplanar orientation with respect to main four ring structure in most cases (Ф3 is near 180^◦ ^or -180^◦^).

## Conclusion

In search of novel antitumoral compounds, a set of 7-aryl-10,11-dihydro-7*H*-chromeno [4,3-*b*] quinoline-6,8(9*H*, 12*H*)-dione derivatives were synthesized by a simple one-pot method using silica sulfuric acid/NaNO_2 _as an oxidative agent for aromatization of 1,4-DHPs. The cytotoxic activity of these compounds was evaluated *in-vitro* on three different cancer cell lines (K562, LS180, and MCF-7). Most of synthetic compounds showed moderate cytotoxic activities. Comparison of the cytotoxic activity of these novel oxidized derivatives with non-oxidized counterpart revealed that oxidation of dihydropyridine ring to pyridine, improves the cytotoxic activity especially in LS180 cell line. Conformational analysis revealed that some conformational aspects of oxidized derivatives such as orientation of C_7_-aryl were clearly different from non-oxidized ones. Therefore, these novel condensed derivatives seem to have promising anticancer properties and further investigation on this group, especially by optimization of heterocyclic and aromatic rings of the structure, could potentially lead to the discovery of potent cytotoxic agents.
